# Characterization of Tauopathy in a Rat Model of Post-Stroke Dementia Combining Acute Infarct and Chronic Cerebral Hypoperfusion

**DOI:** 10.3390/ijms21186929

**Published:** 2020-09-21

**Authors:** Dong Bin Back, Bo-Ryoung Choi, Jung-Soo Han, Kyoung Ja Kwon, Dong-Hee Choi, Chan Young Shin, Jongmin Lee, Hahn Young Kim

**Affiliations:** 1 Department of Neurology, Research Institute of Medical Science, Konkuk University School of Medicine, Seoul 05029, Korea; bicycle0106@gmail.com (D.B.B.); ppo_choi@naver.com (B.-R.C.); kjjasmine@hanmail.net (K.J.K.); 2 Department of Biological Sciences, Konkuk University, Seoul 05029, Korea; jshan06@konkuk.ac.kr; 3 Department of Medicine, Konkuk University School of Medicine, Seoul 05029, Korea; dchoi@kku.ac.kr; 4 Department of Pharmacology, Konkuk University School of Medicine, Seoul 05029, Korea; chanyshin@kku.ac.kr; 5 Department of Rehabilitation Medicine, Konkuk University School of Medicine, Seoul 05029, Korea; leej@kuh.ac.kr

**Keywords:** post-stroke dementia, tauopathy, animal model, chronic cerebral hypoperfusion, glymphatic system

## Abstract

Post-stroke dementia (PSD) is a major neurodegenerative consequence of stroke. Tauopathy has been reported in diverse neurodegenerative diseases. We investigated the cognitive impairment and pathomechanism associated with tauopathy in a rat model of PSD by modeling acute ischemic stroke and underlying chronic cerebral hypoperfusion (CCH). We performed middle cerebral artery occlusion (MCAO) surgery in rats to mimic acute ischemic stroke, followed by bilateral common carotid artery occlusion (BCCAo) surgery to mimic CCH. We performed behavioral tests and focused on the characterization of tauopathy through histology. Parenchymal infiltration of cerebrospinal fluid (CSF) tracers after intracisternal injection was examined to evaluate glymphatic function. In an animal model of PSD, cognitive impairment was aggravated when BCCAo was combined with MCAO. Tauopathy, manifested by tau hyperphosphorylation, was prominent in the peri-infarct area when CCH was combined. Synergistic accentuation of tauopathy was evident in the white matter. Microtubules in the neuronal axon and myelin sheath showed partial colocalization with the hyperphosphorylated tau, whereas oligodendrocytes showed near-complete colocalization. Parenchymal infiltration of CSF tracers was attenuated in the PSD model. Our experimental results suggest a hypothesis that CCH may aggravate cognitive impairment and tau hyperphosphorylation in a rat model of PSD by interfering with tau clearance through the glymphatic system. Therapeutic strategies to improve the clearance of brain metabolic wastes, including tau, may be a promising approach to prevent PSD after stroke.

## 1. Introduction

Stroke is one of the leading causes of death in most developed and developing countries [1]. Post-stroke cognitive dysfunction is one of the most common and severe consequences of stroke [2]. The risk of developing cognitive dysfunction after stroke, including mild cognitive impairment, can be as high as 80% [2]. Because any dementia that develops or becomes aggravated after stroke can be defined as post-stroke dementia (PSD), its clinical manifestations and underlying pathophysiology may be heterogeneous [3]. Strategic infarct dementia or multi-infarct dementia, which develop immediately after stroke, can be considered as early-onset PSD, whereas delayed-onset PSD can develop slowly after stroke in patients with underlying conditions such as previous lacunes or white matter lesions [3]. Considering that white matter lesions induced by chronic cerebral hypoperfusion (CCH) are one of the predisposing factors for PSD, we hypothesized that CCH may play a key role in the development of PSD after stroke. We simulated this clinical hypothesis by performing two different modeling experiments in the same animal by middle cerebral artery occlusion (MCAO) surgery to mimic acute ischemic stroke and bilateral common carotid artery occlusion (BCCAo) surgery to mimic CCH.

In previous studies, including ours, cognitive impairments in rats with permanent BCCAo proved its usefulness as an animal model mimicking vascular dementia [4,5,6]. White matter disintegration in this animal model contributed to the development of cognitive impairment [4,6]. We showed that CCH can provoke PSD after stroke using a rat model with a combination of MCAO and BCCAo [7]. When acute ischemic stroke was combined with CCH, neuroinflammation and amyloid pathology were enhanced in the ipsilateral cortex, thalamus, and hippocampus [7]. We suggested that CCH may contribute to the development of PSD by interfering with amyloid clearance [7].

Tauopathy has been implicated as the main pathophysiology underlying diverse neurodegenerative diseases, including primary age-related tauopathy, progressive supranuclear palsy, corticobasal degeneration, and chronic traumatic encephalopathy [8,9]. In animal studies, hyperphosphorylation of tau has been reported in a mouse model of CCH that was induced by unilateral common carotid artery occlusion [10,11]. The glymphatic system has been reported to be responsible for the clearance of not only amyloid but also other brain metabolic wastes, such as tau and synuclein [12,13,14,15,16]. An impaired glymphatic system has been reported in animal models associated with CCH, including unilateral common carotid artery occlusion in mice [17] and BCCAo in rats [7]. Based on these findings, we hypothesized that tauopathy may be one of the main pathomechanisms for cognitive impairment in a rat model of PSD with acute ischemic stroke and CCH. Here, we investigated the cognitive impairment and pathomechanisms associated with tauopathy in a rat model of PSD.

## 2. Results

### 2.1. Experimental Groups

**Figure 1:**
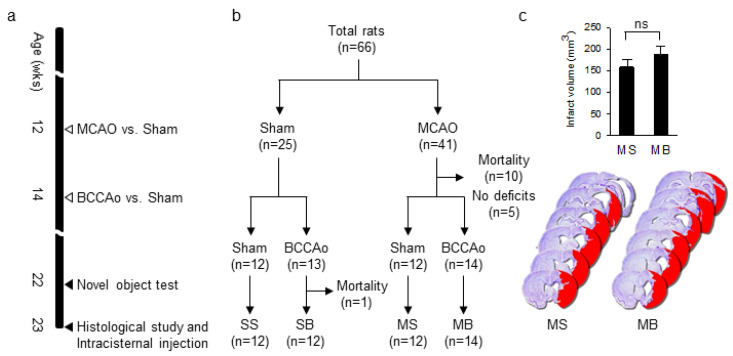
(**a**) The time line of the experiment. (**b**) Allocation of rats into groups. (**c**) Quantification of the infarct volume and representative images of cresyl violet staining in MS and MB rats. n = 6 in both groups; MCAO, middle cerebral artery occlusion; BCCAo, bilateral common carotid artery occlusion; SS, sham + sham; SB, sham + BCCAo; MS, MCAO + sham; MB, MCAO + BCCAo; ns, not significant.

According to the time line of the experiment (Figure 1a), rats were randomly distributed into four groups, based on a 2 × 2 combination of MCAO and BCCAo surgeries (Figure 1b). In detail, the MCAO was either followed by a BCCAo (MB) or a sham BCCAo (MS), and the sham MCAO was followed by either a BCCAo (SB) or a sham BCCAo (SS), with 12 to 14 rats per group (Figure 1b). The mortality rate for MCAO and BCCAo surgeries was 24.4% (10 of 41) and 3.7% (1 of 27), respectively. Based on the results of the modified neurological severity score (mNSS), rats without significant neurological deficits after MCAO surgery (mNSS 0-1) were excluded because of the possibility of no infarct (*n* = 5).

### 2.2. Infarct Volume

Although infarct volume was slightly larger in the MB rats than in the MS rats (n = 6 per group), the difference was not statistically significant (t_(10)_ = −1.107, *p* = 0.294, Figure 1c). Consistent with our previous results [7], the additional BCCAo surgery did not increase the infarct volume of the MCAO surgery.

### 2.3. Cognitive Impairments

**Figure 2:**
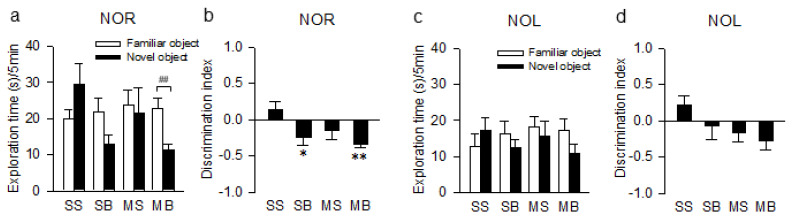
Exploration time and the discrimination index in the novel object recognition test (**a**,**b**) and the novel object location test (**c**,**d**). n = 6–8 per group; NOR, novel object recognition; NOL, novel object location; SS, sham + sham; SB, sham + BCCAo; MS, MCAO + sham; MB, MCAO + BCCAo; * *p* < 0.05 and ** *p* < 0.01 compared to SS; ## *p* < 0.01 using the *t*-test.

For the novel object recognition test (NOR), the exploration time for the novel object compared to the familiar object was significantly decreased in the MB rats (t_(14)_ = 3.483, *p* < 0.01, Figure 2a). The discrimination index was significantly different among groups (F_(3,22)_ = 4.720, *p* < 0.05, Figure 2b). On post hoc analysis, the discrimination index in the SB and MB rats showed an impaired preference for the novel object over the familiar object compared to the SS rats (*p* < 0.01 in MB rats and *p* < 0.05 in SB rats, Figure 2b). For the novel object location test (NOL), the exploration time for the familiar object and the novel object within groups was not statistically different across all groups (Figure 2c). Although the discrimination indexes in the SB, MS, and MB rats compared to the SS rats were all negative, indicating an impaired preference for the novel object over the familiar object, the differences were not statistically significant (F_(3,22)_ = 2.083, *p* = 0.132, Figure 2d).

### 2.4. Tauopathy in the Cortex

**Figure 3:**
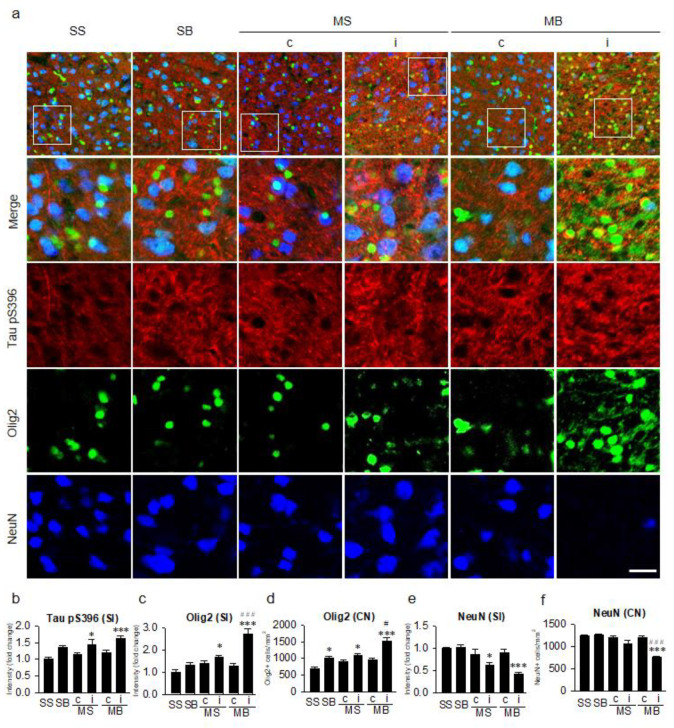
Tauopathy in the cortex. (**a**) Triple-label immunohistochemistry of tau hyperphosphorylation at the serine 396 residue (Tau pS396) in conjunction with oligodendrocytes (Olig2) and neurons (NeuN). Quantitative analysis of the signal intensities of Tau pS396 (**b**), the signal intensities of Olig2 (**c**), the number of Olig2-positive cells (**d**), the signal intensities of NeuN (**e**), and the number of NeuN-positive cells (**f**). n = 6 per group; scale bar = 20 μm; SS, sham + sham; SB, sham + BCCAo; MS, MCAO + sham; MB, MCAO + BCCAo; MCAO, middle cerebral artery occlusion; BCCAo, bilateral common carotid artery occlusion; c, contralateral; i, ipsilateral; SI, signal intensity; CN, cell number; * *p* < 0.05 and *** *p* < 0.001 compared to SS; # *p* < 0.05 and ### *p* < 0.001 compared to the ipsilateral MS.

Tau hyperphosphorylation at Ser396 as measured by signal intensity of Tau pS396 in the cortex (Figure 3a) was significantly different among the groups (F_(5,30)_ = 6.458, *p* < 0.001, Figure 3b). On post hoc analysis, the expression of Tau pS396 was significantly enhanced in the ipsilateral cortex of the MS and MB rats compared to the SS rats (*p* < 0.05 in MS and *p* < 0.001 in MB, Figure 3b). Although ipsilateral tau hyperphosphorylation was higher in the MB rats than in the MS rats, it was not statistically significant between the two groups on post hoc analysis (*p* = 0.640, Figure 3b). Signal intensity of Olig2, which indicated the nucleus and soma of the oligodendrocytes, in the cortex was significantly different among groups (F_(5,30)_ = 17.557, *p* < 0.001, Figure 3c) as well as in their cell number (F_(5,30)_ = 14.765, *p* < 0.001, Figure 3d), with a significant enhancement in the ipsilateral cortex of the MS and MB rats compared to the SS rats on post hoc analysis in the signal intensity (*p* < 0.05 in MS and *p* < 0.001 in MB, Figure 3c) as well as in the cell number (*p* < 0.05 in MS and *p* < 0.001 in MB, Figure 3d). On post hoc analysis, the expression of Olig2 in the ipsilateral cortex of the MB rats was significantly higher than in the ipsilateral cortex of the MS rats in the signal intensity (*p* < 0.001, Figure 3c) and in the cell number (*p* < 0.05, Figure 3d). Neuronal cell loss in the cortex was significantly different among groups in the signal intensity (F_(5,30)_ = 8.948, *p* < 0.001, Figure 3e) as well as in their cell number (F_(5,30)_ = 20.169, *p* < 0.001, Figure 3f). On post hoc analysis, neuronal cell loss was significantly aggravated in the ipsilateral cortex of the MS and MB rats compared to the SS rats in the signal intensity (*p* < 0.05 in MS and *p* < 0.001 in MB, Figure 3e) and in the cell number (*p* < 0.001 in MB, Figure 3f). In addition, neuronal cell number in the ipsilateral cortex of the MB rats was significantly decreased compared to the ipsilateral cortex of the MS rats (*p* < 0.001, Figure 3f).

### 2.5. Tauopathy in the Corpus Callosum

**Figure 4:**
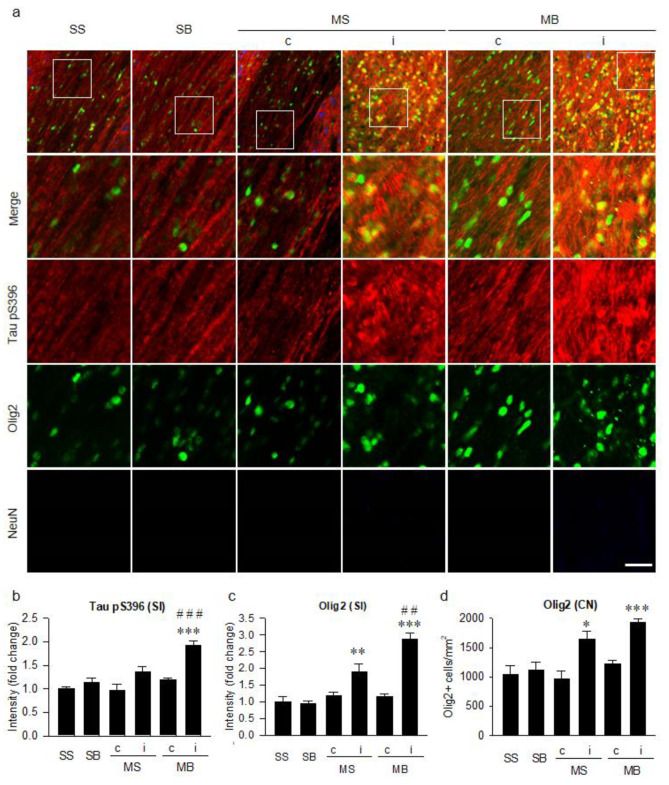
Tauopathy in the corpus callosum. (**a**) Triple-label immunohistochemistry of tau hyperphosphorylation at serine 396 residue (Tau pS396) in conjunction with oligodendrocytes (Olig2) and neurons (NeuN). Quantitative analysis of the signal intensities of Tau pS396 (**b**), the signal intensities of Olig2 (**c**), and the number of Olig2-positive cells (**d**). n = 6 per group; scale bar = 20 μm; SS, sham + sham; SB, sham + BCCAo; MS, MCAO + sham; MB, MCAO + BCCAo; MCAO, middle cerebral artery occlusion; BCCAo, bilateral common carotid artery occlusion; c, contralateral; i, ipsilateral; SI, signal intensity; CN, cell number; * *p* < 0.05, ** *p* < 0.01 and *** *p* < 0.001 compared to SS; ## *p* < 0.01 and ### *p* < 0.001 compared to the ipsilateral MS.

Tau hyperphosphorylation at Ser396 in the corpus callosum (Figure 4a) was significantly different among groups (F_(5,30)_ = 13.346, *p* < 0.001, Figure 4b). On post hoc analysis, the expression of Tau pS396 in the ipsilateral corpus callosum of the MB rats was significantly higher than in the SS rats (*p* < 0.001, Figure 4b). On post hoc analysis, the expression of Tau pS396 in the ipsilateral corpus callosum of the MB rats was still significantly higher than in the MS rats (*p* < 0.001, Figure 4b). Signal intensity of Olig2 in the corpus callosum was significantly different among groups (F_(5,30)_ = 23.761, *p* < 0.001, Figure 4c) as well as in their cell number (F_(5,30)_ = 10.658, *p* < 0.001, Figure 4d), with a significant enhancement in the ipsilateral corpus callosum of the MS and MB rats compared to the SS rats on post hoc analysis in the signal intensity (*p* < 0.01 in MS, *p* < 0.001 in MB, Figure 4c) and in the cell number (*p* < 0.05 in MS, *p* < 0.001 in MB, Figure 4d). On post hoc analysis, the expression in the signal intensity of Olig2 in the ipsilateral corpus callosum of the MB rats was still significantly higher than in the MS rats (*p* < 0.01, Figure 4c). NeuN staining, which was used to detect the soma of the neurons, was not observed in the corpus callosum because of the scarcity of neuronal soma in the white matter (Figure 4a).

### 2.6. Histological Correlation of Tauopathy

**Figure 5:**
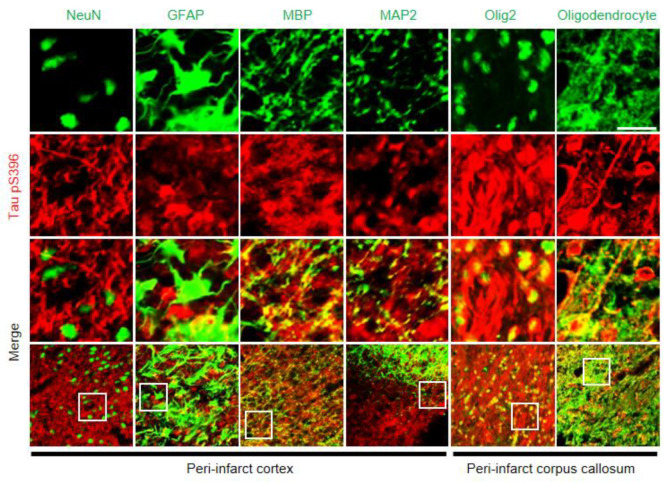
Histological correlation of tauopathy in the peri-infarct area of MB rats. Double-label immunohistochemistry of tau hyperphosphorylation at serine 396 residue (Tau pS396) and various markers, including NeuN, glial fibrillary acidic protein (GFAP), myelin basic protein (MBP), microtubule associated protein 2 (MAP2), Olig2, and Oligodendrocyte. Signals of NeuN, GFAP, MBP, and MAP2 were captured in the peri-infarct cortex and Olig2 and Oligodendrocyte in the peri-infarct corpus callosum. scale bar = 20 μm; MB, MCAO + BCCAo.

To characterize tauopathy in the peri-infarct area of MB rats, we investigated how Tau pS396 expression was correlated with various structures, such as neurons (NeuN), astrocytes (GFAP), the myelin sheath (MBP), microtubules in the neuronal axon (MAP2), nuclei and soma of oligodendrocytes (Olig2), and the whole oligodendrocyte with dendritic processes (Oligodendrocyte) (Figure 5). Intracellular aggregation of hyperphosphorylated tau was colocalized with neuronal axons and oligodendrocytes, but not with astrocytes. Neuronal axons and their surrounding myelin sheaths, as indicated by anti-MAP2 and anti-MBP, were correlated with signals of tau hyperphosphorylation in the peri-infarct cortex. Normal linear patterns of the axons and their myelin sheaths were disrupted. Colocalization of hyperphosphorylated tau with oligodendrocytes was confirmed by two different antibodies, anti-Olig2 and anti-Oligodendrocytes in the peri-infarct corpus callosum.

### 2.7. Parenchymal Infiltration of Cerebrospinal Fluid (CSF) Tracers

**Figure 6:**
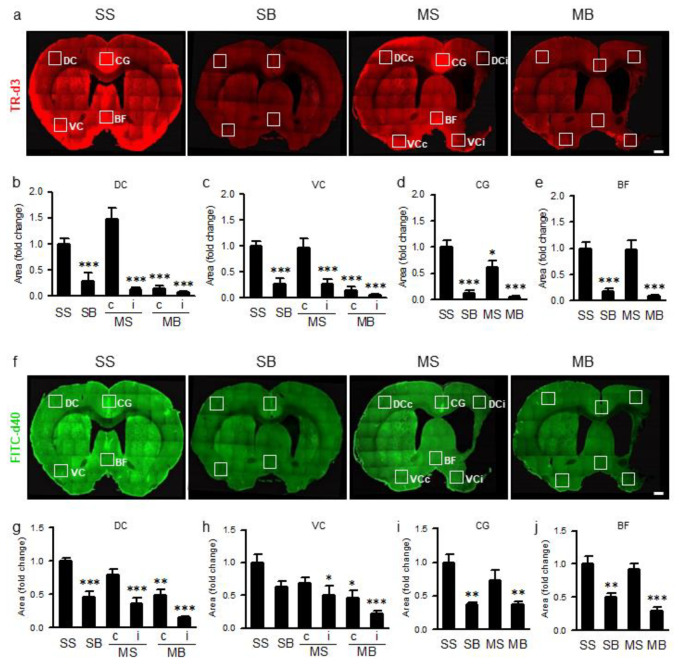
(**a**) Parenchymal infiltration of cerebrospinal fluid tracer (CSF) with Texas Red^®^-conjugated dextran of 3kDa (TR-d3) and its quantification in dorsal cortex (DC, **b**), ventral cortex (VC, **c**), cingulate gyrus (CG, **d**), and basal forebrain (BF, **e**). (**f**) Parenchymal infiltration of CSF tracer with fluorescein isothiocyanate (FITC)-conjugated dextran of 40kDa (FITC-d40) and its quantification in DC (**g**), VC (**h**), CG (**i**), and BF (**j**). n = 6 per group; scale bar = 1 mm; SS, sham + sham; SB, sham + BCCAo; MS, MCAO + sham; MB, MCAO + BCCAo; MCAO, middle cerebral artery occlusion; BCCAo, bilateral common carotid artery occlusion; c, contralateral; i, ipsilateral; * *p* < 0.05, ** *p* < 0.01, and *** *p* < 0.001 compared to SS.

To evaluate glymphatic function indirectly, we investigated parenchymal infiltration of two CSF tracers with different molecular weights. The fluorescent areas of Texas Red^®^-conjugated dextran (TR-d3) (Figure 6a), reflecting parenchymal infiltration of tracers through the glymphatic system, were significantly different among groups in all selected areas of the cerebral hemisphere (F_(5,30)_ = 2.277, *p* < 0.001 in the dorsal cortex, DC; F_(5,30)_ = 16.491, *p* < 0.001 in the ventral cortex, VC; F_(3,20)_ = 23.450, *p* < 0.001 in the cingulate gyrus, CG; and F_(3,20)_ = 18.543, *p* < 0.001 in the basal forebrain, BF; Figure 6b–e). On post hoc analysis, compared to the SS rats, the fluorescent areas of TR-d3 in the DC and VC were significantly decreased in all groups except in the contralateral cortex of the MS rats (Figure 6b,c). In the midline structures, the TR-d3 fluorescent areas in the CG were significantly decreased in the SB, MS, and MB rats (Figure 6d), whereas the TR-d3 fluorescent areas in the BF were significantly decreased in the SB and MB rats (Figure 6e). Fluorescein isothiocyanate (FITC)-conjugated dextran (FITC-d40) infiltration (Figure 6f) was also significantly different among groups in all selected areas of the cerebral hemisphere (F_(5,30)_ = 16.283, *p* < 0.001 in DC; F_(5,30)_ = 6.590, *p* < 0.001 in VC; F_(3,20)_ = 10.811, *p* < 0.001 in CG; and F_(3,20)_ = 16.316, *p* < 0.001 in BF; Figure 6g–j). On post hoc analysis, compared to the SS rats, the fluorescent areas of FITC-d40 in the DC were significantly decreased in all groups except in the contralateral cortex of the MS rats (Figure 6g), whereas the fluorescent areas of FITC-d40 in the VC were significantly decreased in the ipsilateral cortex of the MS rats and the bilateral cortices of the MB rats (Figure 6h). FITC-d40 fluorescent areas in the CG and BF were significantly decreased in the SB and MB rats compared to those in the SS rats (Figure 6i,j).

## 3. Discussion

In a previous animal study, we suggested that CCH may be a provoking factor for the development of PSD after stroke [7]. Spatial memory in the water maze task was synergistically impaired when BCCAo was combined with MCAO [7]. The MB rats, which underwent a combination of MCAO and BCCAo, simulated the clinical development of PSD, which is similar to the development of stroke in patients with white matter lesions possibly induced by underlying CCH. In the present study, MB rats showed an impaired preference in the novel object as compared with the SS rats in the performance of the NOR test. Although the results of the novel object tests were not as strong as those of the water maze task in our previous study [7], it showed some tendency for cognitive impairments in MB rats. Taken together, we could state that our PSD animal model developed some cognitive impairment. However, the infarct volume was not different between the MS and MB rats. These experimental findings are consistent with the clinical manifestations, where some stroke patients develop PSD, and some do not, despite having similar stroke volumes [3]. Factors other than stroke volume, such as post-stroke neurodegeneration in various regions related to the infarct, may be more critical in triggering PSD. In our previous study, amyloid pathology was synergistically enhanced in the ipsilateral cortex, thalamus, and hippocampus when BCCAo was combined with MCAO [7]. Neuroinflammation and depolarization of the aquaporin 4 (AQP4) water channel, known to be involved in impairment of the glymphatic system, were associated with enhanced amyloid pathology and cognitive impairment in our previous study using the same animal model of PSD [7]. In the present study, we investigated whether tauopathy can be another culprit involved in the neurodegenerative pathomechanisms associated with PSD.

In various neurodegenerative diseases, tauopathy has been reported as the main pathomechanism of neurodegeneration [8,9]. In animal models of cerebral ischemia, the hyperphosphorylation of tau has been reported in acute ischemic stroke [18,19] and cerebral hypoperfusion [10,11]. Based on these findings in the literature, we hypothesized that tauopathy may have a pathological role in the PSD animal model that combined MCAO and BCCAo. Considering that the ischemia-induced hyperphosphorylation of tau could promote proteolysis of the neuronal cytoskeleton and undermine the stability of microtubules [20], the disruption of axonal transport by tau hyperphosphorylation may be the main pathophysiology of the neurodegeneration in our PSD animal model. Therefore, hyperphosphorylation of axonal tau at the paired helical filaments-1 epitope (pS396) was considered for the evaluation of pathological hyperphosphorylation of tau in our study. However, further studies are needed that will focus on the various epitopes other than pS396 epitope [20]. Although we measured tau hyperphosphorylation at pS396 epitope based on the results of immunohistochemistry, quantitative methods, such as Western blotting, are needed to independently confirm the results. In addition, other post-translational modifications of tau, including glycosylation, glycation, prolyl-isomerization, cleavage or truncation, nitration, polyamination, ubiquitination, sumoylation, and oxidation and aggregation, need further attention [21].

As reported in the literature, we found that tau hyperphosphorylation was increased after either MCAO or BCCAo surgery and was most significantly enhanced in the peri-infarct area of MB rats where the effects of the two surgeries were combined. On post hoc analysis, an additional significant increase of tau hyperphosphorylation in the peri-infarct area of MB rats compared to that of the MS rats was more prominent in the corpus callosum than in the cortex. Well-aligned linear axonal structures in the corpus callosum were disrupted and conglomerated with enhanced tau hyperphosphorylation in the peri-infarct area. The abundance of neuronal axons and supporting oligodendrocytes in the white matter may explain the more prominent tau hyperphosphorylation in the corpus callosum. Oligodendrocytes, the main glial cells in the white matter, have been reported to be vulnerable to ischemic insults [22,23]. In accordance with these findings, the white matter may be more vulnerable to tauopathy in our PSD animal model.

Tauopathies have been reported in diverse cell types, including neurons, oligodendrocytes, and astrocytes [24,25]. In our experiment, tau hyperphosphorylation was mainly colocalized with neurons and oligodendrocytes, but not with astrocytes. These findings are consistent with those of previous studies in the literature, which reported that tau-positive cells are mainly correlated with oligodendrocytes and not with astrocytes in the ipsilateral white matter of rats with MCAO [26,27]. The increase in the number of Olig2-positive cells has been reported in various brain injury models, including stab wound, MCAO, and amyloid injection through diverse mechanisms, such as transcriptional upregulation, oligogenesis, cell proliferation, and recruitment of Olig2-positive progenitor cells to the injury sites [28]. Upregulation of oligodendrocyte progenitor cells has also been reported in animal models during the recovery phase after acute infarcts or long-term cerebral hypoperfusion [29,30]. As previously reported in the literature [28,31], the increased expression of Olig2 in our experiment, indicating all oligodendrocyte lineage cells, suggests that some oligodendrocyte progenitor cells may be maturing into oligodendrocytes and may be indicative of an enhanced restorative process after ischemic injury in MB rats. In addition, more neuronal cell loss in the peri-infarct cortex might also have contributed to the cognitive impairments in the MB and the MS rats as compared to the SS rats. Moreover, neuronal cell loss, measured in their cell number, observed in the peri-infarct cortex, was more accentuated in the MB rats than in the MS rats. More neuronal cell loss in the peri-infarct cortex might also have contributed to more cognitive impairment in the MB rats than in the MS rats

The glymphatic system has been proposed as a brain metabolic waste clearance mechanism comprised of a para-arterial CSF influx, trans-parenchymal interstitial fluid (ISF) bulk flow, and para-venous ISF clearance [12,13,14,15,16]. Metabolic waste proteins, such as amyloid, tau, and synuclein, can be removed by the glymphatic system, and its impairment can lead to pathological accumulation of metabolic wastes that lead to neurodegenerative diseases, such as Alzheimer’s disease, Parkinson’s disease, and traumatic brain encephalopathy [12,13,14,15,16]. The hypothesis that cerebral arterial pulsation is a driving force that maintains the ISF bulk flow has been supported by experimental findings showing that the glymphatic system was impaired by unilateral carotid ligation in mice [17]. In our experiment, the permanent ligations of both common carotid arteries may have attenuated the arterial pulsation and deteriorated the influx of fluorescent CSF tracers. Parenchymal infiltration of CSF tracers was bilaterally attenuated in the SB and MB rats, which underwent BCCAo surgery. MCAO surgery performed in the MS and MB rats attenuated the parenchymal infiltration of CSF tracers in the ipsilateral cortex but not in the contralateral cortex. These findings suggest that the glymphatic system may be impaired globally by BCCAo or ipsilaterally by MCAO. Perturbation of the AQP4 water channel has been suggested as a possible mechanism to explain the impaired glymphatic system [7,8,12,17,32,33,34]. Change in the AQP4 distribution from a perivascular to a parenchymal pattern, also known as the depolarization of AQP4, has been reported in various animal [8,12,17,32] and human studies [33,34] of various neurodegenerative diseases. In our previous study, using the same animal model of PSD as the present study, a similar change in AQP4 distribution was observed [7]. Although the pathomechanisms of tauopathy in neurodegeneration have not been fully elucidated, the phosphorylation theory of tauopathy is based on diverse processes, including tau phosphorylation, proteolysis, oligomerization, and aggregation that leads to the formation of paired helical filaments and neurofibrillary tangles [24]. During these processes, toxic tau oligomers can be released into the interstitial space [24,35], carried by the ISF bulk flow, and cleared through the glymphatic system.

Although further studies are needed to confirm the causal relationship, our study results suggest the hypothesis that synergistic increases of tauopathy in the peri-infarct area of MB rats may be associated with enhanced tau hyperphosphorylation not only due to the ischemic injury but also due to the impaired clearance of interstitial tau oligomers. Therapeutic strategies to improve the clearance of brain metabolic wastes, including tau, may be a promising approach to prevent PSD after stroke.

## 4. Materials and Methods

### 4.1. Animals

Male Wistar rats (300–350 g), aged three months, were used for this study (Orient Bio, Seoul, Republic of Korea). Animals were housed under standard vivarium conditions, with a temperature of 22 ± 1 °C, 50 ± 10% humidity, a 12:12 h light-dark cycle, and ad libitum access to food and water. All animal experiments were performed in accordance with the guidelines of the Institutional Animal Care and Use Committee (IACUC) of Konkuk University (approval number KU17124 and approved on 16 Oct 2017 by IACUC) and the Animal Research: Reporting of In Vivo Experiments (ARRIVE) guidelines (https://www.nc3rs.org.uk/arrive-guidelines).

### 4.2. Experimental Design

Rats were subjected to sequential surgeries to generate the PSD model, as described in our previous study [7]. The initial MCAO surgery was followed two weeks later by a BCCAo surgery (Figure 1a). The time interval between two surgeries was set as 2 weeks to investigate how CCH, induced by BCCAo during the recovery phase after the acute ischemic response, could contribute to the development of PSD. In our previous study [7], we titrated the time interval as 2 weeks to minimize mortality and maximize the pathological interaction. The MCAO surgery was performed using the standard intraluminal suture approach for 90 min. The BCCAo was accomplished by a permanent double-ligation of the bilateral common carotid arteries using silk sutures. The rats were divided into four groups based on a 2 × 2 combination of MCAO and BCCAo surgeries (Figure 1b). In detail, the MCAO was either followed by a BCCAo (MB) or a sham BCCAo (MS), and the sham MCAO was followed by either a BCCAo (SB) or a sham BCCAo (SS). All rats were anesthetized using a 5% isoflurane/oxygen mix and maintained with 3% isoflurane/oxygen throughout the surgeries. The body temperature of the rats was maintained as 37 ± 0.5 °C using a heating pad. Novel object tests were performed 10 weeks after the initial surgery. Rats were sacrificed for histological evaluation after all behavioral experiments were performed. Rats were coded with numbers, and all investigators were blinded to the treatment groups until the end of the data analysis. The timeline of the experiments is shown in Figure 1a.

### 4.3. Neurological Function Test

The mNSS, ranging from 0 to 14, is a composite scoring system that assesses neurological function by evaluating the motor status, abnormal movements, sensory abilities, and reflexes of the rats [36]. One point is awarded for the inability of the rat to perform each individual task correctly.

### 4.4. Novel Object Tests

The novel object test was conducted according to the methods described in our previous study [6]. Briefly, the rats were placed in a 40 cm × 40 cm × 40 cm box and were allowed to explore the empty area for 10 min/day for a 5-day habituation period. Twenty-four hours after finishing the last habituation phase, the rats were exposed for 5 min to two identical objects that were placed in the test area. After a 3-h interval, one of the objects was either re-located (NOL) or replaced by another object (NOR), and the rats were allowed to explore them once more for 5 min. Object exploration was defined as when the center of the rat’s head was oriented within 45° and 4 cm of the object. Climbing over or sitting on an object was not considered an exploration. Exploratory behaviors were videotaped and analyzed in a blind manner. The exploration time of the novel object and the familiar object for 5 min was measured. The discrimination index was calculated as follows: [(time observing novel) − (time observing familiar)]/[(time observing novel) + (time observing familiar)] × 100.

### 4.5. Tissue Preparation

After rats were anesthetized with isoflurane and transcardially perfused with 0.01 M phosphate-buffered saline (PBS) followed by 4% paraformaldehyde (PFA), the brains were removed, post-fixed overnight in the same fixative, successively cryoprotected in 30% sucrose solution, and embedded in an optimal cutting temperature compound. The brains were serially sliced into 30-μm coronal sections on a cryostat for cresyl violet staining or immunohistochemistry.

### 4.6. Cresyl Violet Staining and Infarct Volume

Sections were mounted onto resin-coated glass slides, dried for 10 days, hydrated through descending concentrations of ethanol, and finally dipped twice in distilled water. Sections were immersed in cresyl violet acetate (Sigma Aldrich, St. Louis, MI, USA) dissolved at 0.5% (*w/v*) in distilled water for 5 min, and then dehydrated through ascending concentrations of ethanol. Finally, the sections were defatted in xylene and coverslipped with Permount™ mounting medium. The infarct volume was calculated using six representative cresyl violet-stained slices selected at 2-mm intervals in the MS and MB rats (n = 6 per group).

### 4.7. Immunohistochemistry

All sections were washed (1x PBS/0.3% Triton X-100) and incubated in blocking serum (1x PBS/10% normal donkey serum/0.3% Triton X-100). Triple-label immunohistochemistry was conducted to compare the alterations in tau phosphorylation at serine 396 residue in correlation with oligodendrocytes and neurons. Accordingly, sections were stained in a solution (1x PBS/0.15% normal donkey serum/0.3% Triton X-100) containing primary antibodies against Tau pS396, oligodendrocytes, and neurons and incubated overnight at room temperature. Double-label immunohistochemistry of Tau pS396 and various markers of either neurons, astrocytes, or oligodendrocytes was performed. The primary antibodies were employed as follows: rabbit anti-Tau pS396 (1:200, Abcam, Cambridge, UK), mouse anti-Olig2 (1:500, EMD Millipore, Burlington, NJ, USA), guinea pig anti-NeuN (1:500, EMD Millipore), mouse anti-glial fibrillary acidic protein (GFAP) (1:1000, BD Bioscience, San Jose, CA, USA), chicken anti-myelin basic protein (MBP) (1:500, Thermo Fisher Scientific, Waltham, MA, USA), mouse anti-microtubule associated protein 2 (MAP2) (1:500, Sigma-Aldrich), and mouse anti-Oligodendrocytes (1:500, EMD Millipore). The secondary antibodies diluted 1:200 in the washing buffer were used as follows: donkey anti-rabbit Alex Fluor 568, donkey anti-mouse Alexa Fluor 488, goat anti-guinea pig Alexa Fluor 633, and goat anti-chicken Alexa Fluor 647 (all from Invitrogen, Carlsbad, CA, USA). Stained sections were mounted on resin-coated slides and dried for 30 min. Slides were then coverslipped with ProLong^®^ Gold antifade reagent (Invitrogen).

### 4.8. Intracisternal Injection of CSF Tracers

We used 0.5% solutions of a small molecular weight (3 kDa) Texas Red^®^-conjugated dextran (TR-d3, Thermo Fisher Scientific) and a large molecular weight (40 kDa) fluorescein isothiocyanate (FITC)-conjugated dextran (FITC-d40, Sigma-Aldrich) in a 1:1 ratio dissolved in the artificial CSF (aCSF, Tocris Bioscience, Bristol, UK) for the co-infusion experiment [32]. To prevent leakage of the CSF tracers, the cannula was tightly arranged in a series as follows: A Hamilton 700 series syringe with a 22-gauge needle (Sigma-Aldrich) was connected to PE50 and PE10 polyethylene tubing. The other end of the PE10 tubing was linked to a 28-gauge injection cannula inserted into the cisterna magna, through which the CSF tracers moved. The rats were initially induced with 3% isoflurane and then anesthetized intraperitoneally with a combination of Zoletil^®^ 50 (30 mg/kg) and xylazine (10 mg/kg). Anesthetized rats were fixed in a stereotaxic frame while the atlanto-occipital membrane overlying the cisterna magna was surgically exposed. A total volume of 70 μL of the CSF tracers was infused at a rate of 1.6 μL/min using a syringe pump. After the infusion was finished, the needle was left in place for 30 min. Then, the anesthetized rats were transcardially perfused and fixed with 4% PFA. Fluorescence imaging was performed on 30 μm coronal sections of the brain tissue.

### 4.9. Quantitative Analysis

The fluorescence signal intensity of Tau pS396, Olig2, and NeuN immunoreactivity was evaluated from 30 μm coronal brain sections (bregma 0.36 mm, 3 sections per rat, n = 6 per group). Images of a region of interest (ROI) encompassing the cortex and corpus callosum were captured using a laser scanning confocal microscope (Olympus FV1000, Tokyo, Japan) with a 10× objective. The ipsilateral ROIs of the MS and MB rats were selected in the peri-infarct area as reported previously in the literature [29], and contralateral ROIs were selected as symmetrical regions in the contralateral hemisphere. The signal intensity in the ROIs was measured using FluoView software (Olympus), averaged, and expressed as changes in relative percent compared to the SS rats. The number of Olig2- or NenN-positive cells were counted and averaged per unit area in the same ROIs using the Image J (NIH).

Brain parenchymal infiltration of CSF tracers was imaged from 30 μm coronal brain sections (bregma 1.56 to −3.24 mm, 5 sections per rat, n = 6 per group) using laser scanning confocal microscopy (Olympus FV1000). Images of the ROIs in DC, VC, CG, and BF were captured using a 20× objective and quantified using the Image J (NIH). To evaluate glymphatic function more clearly, we added CG and BF, where the influx of fluorescent CSF tracers is strong, as the ROIs in addition to DC and VC. To evaluate differences between the two CSF tracers, fluorescence channels were split into red or green to separately measure the area covered by each of the co-infused CSF tracers (TR-d3 in red and FITC-d40 in green). Images were converted to 8-bit grayscale and adaptively subtracted in a blind manner. The coverage area above the arbitrary threshold was measured and calculated as the change in relative percent compared to SS rats.

### 4.10. Statistical Analysis

A one-way ANOVA followed by a post hoc Tukey’s honest significant difference test was used to analyze differences in discrimination index, immunohistochemistry quantification, and infiltration of the CSF tracers among groups. Infarct volumes between the MS and MB rats and the simple comparison of exploration time for the familiar and novel objects within groups were analyzed using a two-tailed unpaired Student’s *t*-test. The data are presented as the mean ± standard error of the mean (mean ± SEM). A value of *p* < 0.05 was considered to be statistically significant. Data analyses were performed using the SPSS Statistics 25.0 (IBM, Armonk, NY, USA).

## Abbreviations

PSD	Post-stroke dementia
CCH	Chronic cerebral hypoperfusion
MCAO	Middle cerebral artery occlusion
BCCAo	Permanent occlusion of bilateral common carotid arteries
AQP4	Aquaporin 4
MB	MCAO + BCCAo
MS	MCAO + sham BCCAo
SB	Sham MCAO + BCCAo
SS	Sham MCAO + sham BCCAo
NOR	Novel object recognition test
NOL	Novel object location test
GFAP	Glial fibrillary acidic protein
MBP	Myelin basic protein
MAP2	Microtubule associated protein 2
CSF	Cerebrospinal fluid
DC	Dorsal cortex
VC	Ventral cortex
CG	Cingulate gyrus
BF	Basal forebrain
ISF	Interstitial fluid
FITC	Fluorescein isothiocyanate
ROI	Region of interest
PBS	Phosphate-buffered saline
PFA	Paraformaldehyde
SI	Signal intensity
CN	Cell number
